# Evaluation of an e-learning platform promoting electronic personal health record competence: a pilot trial in older adults

**DOI:** 10.1186/s12889-025-22242-0

**Published:** 2025-03-15

**Authors:** Luis Perotti, Oskar Stamm, Drin Ferizaj, Michael Dietrich, Ilona Buchem, Ursula Müller-Werdan

**Affiliations:** 1 Department of Geriatrics and Medical Gerontology, Charité – Universitätsmedizin Berlin, Corporate Member of Freie Universität Berlin and Humboldt-Universität Zu Berlin, Berlin, 13347 Germany; 2 German Research Center for Artificial Intelligence Berlin, 10559 Berlin, Germany; 3 Berlin University of Applied Sciences, 13154 Berlin, Germany

**Keywords:** Electronic personal health record, Personal health record, Patient portal, E-learning, Older adults, Digital competencies, EHealth literacy, EPA, Germany

## Abstract

**Background:**

Electronic personal health records (ePHRs) play a key role in the digitalization of healthcare, but older adults, often less familiar with digital tools, face access challenges. This study assesses the effects of an interactive, microlearning-based e-learning platform on improving older adults’ ePHR competencies.

**Methods:**

To examine the effects of e-learning platform use on competencies, a pilot trial was conducted with two study groups. One group consisted of young-old adults (YOA) aged 50 to 64 years, and the other of older adults (OA) aged 65 years and older. Participants were recruited via senior organizations and facilities, newsletters and an internal database. Both groups used the learning platform for one week. Participants’ ePHR knowledge (12-item questionnaire) and usage skills (completion time for three ePHR tasks) were measured pre- and post-intervention on site. The intention to use (ITU) the ePHR was surveyed using a Technology Usage Inventory subscale. The usability of the platform was assessed using the System Usability Scale.

**Results:**

Twenty-eight participants (mean age YOA = 56.86, OA = 75.15 years) completed the study, with more women in both groups (YOA: 78.57%, OA: 57.14%). Knowledge improved significantly in both groups: OA increased their median correct answers from 7.00 to 9.00 (*p* = .019, *r* = .63), YOA increased from 7.00 to 10.00 (*p* = .001, *r* = .86). Median task completion times also decreased for both groups: OA from 746.50 to 539.00 s (*p* = .002, *r* = .82), YOA from 487.00 to 351.00 s (*p* = .012, *r* = .67). There were no significant differences between groups in terms of knowledge (*p* = .125) or skill acquisition (*p* = .144). Across the entire population, median ITU scores decreased from 282.00 to 262.00 (*p* = .038, *r* = .39), indicating a reduced intention to use the ePHR, though no changes were observed within groups. The platform’s usability scored a mean of 64.04, suggesting high marginal usability.

**Conclusion:**

Both OA and YOA improved their ePHR competencies after using the learning platform, with no significant differences between groups. The findings suggest that e-learning can enhance ePHR competence in older adults, though improvements in platform usability are needed for wider application in future studies.

**Trial registration:**

German Clinical Trials Register (registration number: DRKS00031730), registered on 20/04/2023—prospectively registered.

**Supplementary Information:**

The online version contains supplementary material available at 10.1186/s12889-025-22242-0.

## Background

While demographic changes like increased longevity are putting pressure on healthcare systems, with more complex, expensive care needs, empowering patients through digital tools like electronic Personal Health Records (ePHRs) can help enable better management of care across sectors [1]. As people age, they tend to utilize healthcare services more through increased medication use and doctor visits [2]. ePHRs empower patients to take control of their health data. Generally, ePHRs are defined as electronic records that contain a patient’s health information, which can be accessed and managed by the patient [3]. Due to this patient-centered approach, ePHRs are considered a paradigm shift, highlighting the sovereignty and autonomy of the patient [4]. ePHRs allow individuals to manage their own health information, including medical history, medication, allergy data, family information and laboratory test results [3]. Furthermore, ePHRs enable patients to access their health information from anywhere, at any time, using secure digital private environments, and the rights of access are managed by the patient [3, 4]. The utilization of the ePHR is associated with several benefits. ePHRs can help to reduce medical errors through greater access to health information [3, 4], improve communication between patients and healthcare providers [5] as well as quality of care [4, 6] and increase patient engagement and empowerment as regards their own care [3, 5, 7]. Additionally, ePHRs can reduce the time spent on information retrieval, allowing clinicians to spend more time on treatment-related tasks [3, 4, 8] and help avoid double examinations or treatments [9].

In Germany, the electronic patient file (elektronische Patientenakte; ePA) was introduced as a national ePHR system. Since 2021, statutory health insurance providers must offer the ePA to their insured members free of charge [9]. Similar to the previous definition of ePHRs, the ePA enables comprehensive storage of medical findings, past examinations, diagnoses and treatments across practices and hospitals. Crucially, the ePA prioritizes patient decision-making authority and empowerment by allowing individuals to control if and how their data are uploaded, stored, deleted and shared with healthcare providers [9, 10]. However, adoption of ePHRs, including the ePA, remains startlingly low, despite the potential benefits for both clinicians and patients [11–14]. In Germany, less than 1% of insured individuals had registered an ePA as of early 2023, and adoption actually halved over the course of 2022, with 170,000 individuals creating an ePA file in the first half of the year and only 84,000 in the second half [15]. There are currently just under one million registered ePHRs in Germany [16].

Several barriers hinder adoption, such as concerns over privacy and security, limited accessibility and digital competence, insufficient technical and social support, perceived complexity, lack of perceived value and general unawareness of novel eHealth technologies, as well as personal and socioeconomic factors [3, 6, 17, 18]. These hurdles disproportionately impact older adults (OA) with less technological experience and self-efficacy [18, 19]. Indeed, feeling inadequate or not regarding technologies as useful can undermine their usage. At a basic level, many are simply unaware ePHRs even exists [20].

To overcome these barriers, conceptual technology training programs that target specific groups such as OA offer a promising solution for those with basic smartphone/computer knowledge [20–22]. Such training programs provide opportunities for users to build familiarity and confidence with complex technological systems through hands-on practice. Digital training strengthens self-efficacy, knowledge and competence, factors that are vital for the sustained use of eHealth technologies long-term [18, 22]. In this domain, digital literacy education, in particular, aims to cultivate perceptions of self-efficacy and competence, core determinants of both initial and continued engagement with eHealth solutions [4, 20, 23] as well as ePHR systems over time [10, 23]. Baartman and de Bruijn [24] consider competence to be defined by the factors of skill, knowledge and attitude. In order to promote the competence of users in dealing with the ePHR, all three dimensions must, therefore, be addressed by suitable interventions. Digital competence is often self-reported rather than objectively tested, potentially misrepresenting OA’ actual ePHR skills. While skills and system knowledge are crucial for effective ePHR use, sufficient facilitative support remains important. The provision of manuals, technical assistance, social support and training sessions can achieve this [17]. A recent meta-analysis by Dong et al. [25] assessed digital health literacy interventions for OA. However, only seven studies were included in the meta-analysis, showing the need for further research to develop practical and effective eHealth interventions for OA. ePHRs are expected to play an increasingly important role in the self-management of disease and care for OA [26]. Therefore, it is of utmost importance to empower OA to independently use ePHRs and enhance their competence and knowledge in using ePHRs through specifically tailored training programs. For this reason, the following study will examine knowledge gains and increases in skill among people aged 50 years and over as the primary endpoint, following use of the high-fidelity prototype of the e-learning platform, ePA Coach.

Prior work has tested its interface and usability, but the actual impact on competence remains unknown [10]. A qualitative study was conducted to gain deeper insight into the needs related to the layout and design of the learning platform ePA Coach [27]. Participants expressed a preference for a simple and clear presentation of information. In a second evaluation phase, older adults were asked to assess the usability of a prior version of the platform, reporting a mean score of 70.41 for the System Usability Scale (SUS) in the online group and 67.97 in the face-to-face group [28]. By using the learning platform, users can acquire the content and skills needed for sovereign use of the German ePHR by both young-old adults (YOA, ages 50–64 years) and OA (ages 65 + years). As secondary endpoints of this study, in addition to testing the usability of the website, the study will explore how the use of the learning platform impacts the intent to use the ePHR in the future. The research presented here forms part of the project “ePA Coach—Digital Sovereignty and the Electronic Health Record”, a project funded by the German Federal Ministry of Education and Research. The following research questions will be determined:

Primary research question:

- RQ1: Does one week of ePA Coach use influence knowledge and skills for ePHR usage among participants?

Secondary research questions:

- RQ2: Are the effects on knowledge and skills, required for the competent use of the ePHR, different between older adults and young-old adults?
- RQ3:How does ePA Coach platform use impact the intention to use the German ePHR among participants?
- RQ4: What were the trends of usage behavior on the ePA Coach platform and how do these relate to knowledge and skill development?
- RQ5: How do older adults and young-old adults rate the usability of the ePA Coach learning platform and how does the perceived usability influence the study outcomes?

## Methods and materials

### Trial design

This pilot trial aims to compare the effect of the ePA Coach learning platform on participants within different age groups. We, therefore, conducted a trial with two planned subgroups, including either participants aged between 50 and 64 years or above 65 years. The choice of the age range of 50 to 64 years for one study arm and 65 and above for the second study was based on an age subdivision often found in the literature [29, 30]. Individuals in the age range of 50 to 64 years are often referred to as “young-old” adults [31]. In a medical geriatric context, OA from the age of 65 years are referred to as “older patients” or “OA” [32]. This categorization was used in this study. We were especially interested in the potential increase in knowledge of the ePHR and skill in handling the ePHR after the use of the learning platform. We based the selection of our sample size in this pilot trial on the considerations of Moore et al. [33] and Julious [34]. We, therefore, set a minimum recruitment goal of 12 subjects for each arm, to which we added approximately 25% of dropouts, based on our experience of studies involving the target group of OA. Both study groups completed the same assessments and received equal intervention. The study included two face-to-face appointments, one visit at baseline (visit 1) and one follow-up visit (visit 2) after the intervention period. The intervention period included the use of the learning platform, ePA Coach (a high-fidelity prototype developed as part of the research project) for one week on the participants’ own devices (PC, smartphone or tablet) and in their own homes. In accordance with the intention-to-treat principle, all participants were free to use the learning platform as often as they wished within the one-week intervention period. We registered this study in the German Clinical Trials Register (registration number: DRKS00031730, registered on 20/04/2023) and obtained approval from the Ethics Committee of the Charité—Universitätsmedizin Berlin (application number: EA1/038/23). The participants did not receive an incentive or reward for completing the study.

### Screening

Prior to the start of the study, telephone screening was conducted to determine which respondents were suitable for the study. In addition, during this telephone screening, the interested individuals were informed in detail about the study purpose and any planned procedures, and questions that arose were answered. The inclusion and exclusion criteria were checked. Moreover, the standardized telephone questionnaire, Telephone Interview for Cognitive Status (TICS) [35], was used to assess cognitive status, in order to identify potential dementia and severe cognitive impairment symptoms. These were exclusion criteria since we identified cognitive impairment as a confounder for learning success. Subsequently, an appointment was made for the baseline assessment.

### Inclusion and exclusion criteria

The following inclusion criteria applied to the study:

#### Inclusion criteria

- 50–64 years of age (study arm, YOA)
-  ≥ 65 years (study arm, OA)
- Internet access at home
- Availability of a device to access websites
- Ability to read and understand German written and spoken language

#### Exclusion criteria for both groups (YOA and OA)

- Participation in the previous study—ePA Coach Intermediate Test 2 (ECZ2)
- Cognitive impairment (TICS ≤ 20: moderately to severely impaired)
- Sensory and/or motor deficits that prevent the use of websites or completion of online questionnaires (self-reported)
- Having a legal guardian (self-reported)

### Study procedure

In order to optimize the organizational and practical procedure of the study, two separate pilot tests were conducted in advance with the subjects of the target group. For recruitment purposes, we contacted potential participants using multipliers (senior organizations and facilities, as well as newsletters) or via the internal database of the Geriatrics Research Group (contacting individuals who have given consent to be contacted for research purposes).

At the beginning of the baseline visit, the procedure of the study was explained to the participants and an overview of the study’s objectives was given. After signing the informed consent form, a questionnaire was filled out to collect participants’ basic characteristics. To measure the change in knowledge of our participants, a single-choice questionnaire was conducted requesting general information about the ePHR. A practical skills test, using ePHR mockups, was conducted on a laptop to assess the participants’ skills in using the ePHR before the intervention. To measure the attitude of participants toward the ePHR, we conducted the Intention To Use (ITU) subscale of the Technology Usage Inventory (TUI) by Kothgassner [36]. Since the usage rates of the ePHR in Germany were quite low, assessing the actual attitude of participants was not feasible. We, therefore, used the ITU subscale as an estimation for future acceptance. After completing the baseline assessments, the participants received a printed link to the ePA Coach learning platform as a handout. An initial registration and login on the learning platform were performed directly on-site, with the assistance of study staff. The username (randomly generated ID) and password for registration and login were assigned to the participants and handed out as a separate handout.

Within the intervention period of one week, the participants had the opportunity to use the learning platform ePA Coach. The platform was to be used online at the participants’ own home. Following the intention-to-treat principle, the completion of all available learning units was not mandatory for participation in visit 2.

As part of the follow-up visit after one week, validated assessments of the TUI, subscale ITU, SUS [37] and the Chatbot Usability Questionnaire (CUQ) [38] were carried out. Furthermore, to determine the change in knowledge of the ePHR after the intervention, a single-choice questionnaire was conducted again. To measure the increase in skill in operating the ePHR, the participants conducted a second skills test.

### Allocation, randomization and blinding

**Fig. 1:**
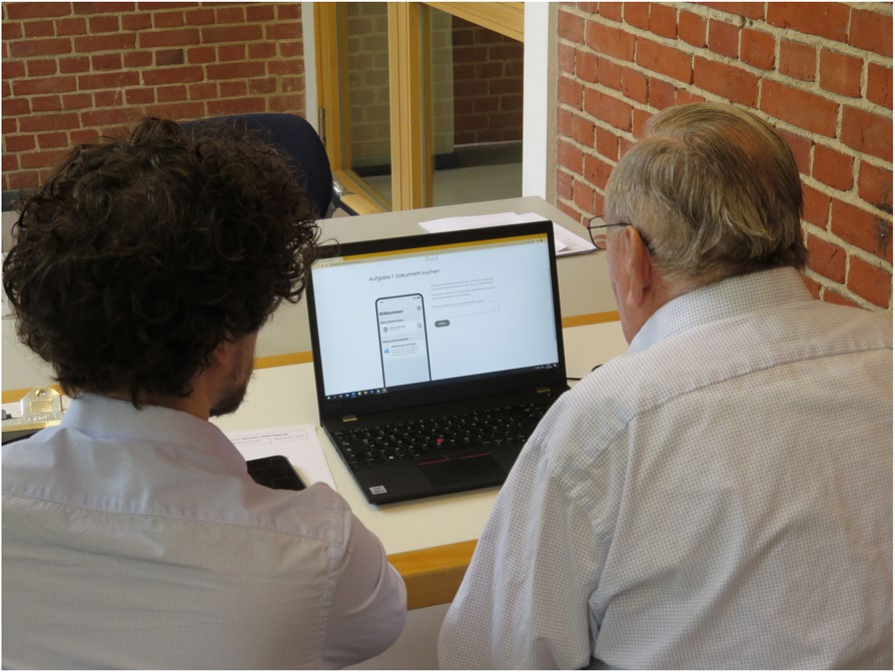
Study participant during the skills test (right) and observation by study staff member (left)

The study did not include an allocation of participants to study arms. We conducted a planned subgroup analysis based on age (between 50 and 64 years or above 64 years), with efforts made to ensure equal group sizes. Since all participants were assigned to one of the two groups based on their age, no measures for blinding were applicable, and no further stratification was performed (Fig. 1).

### The ePA Coach learning platform

**Fig. 2:**
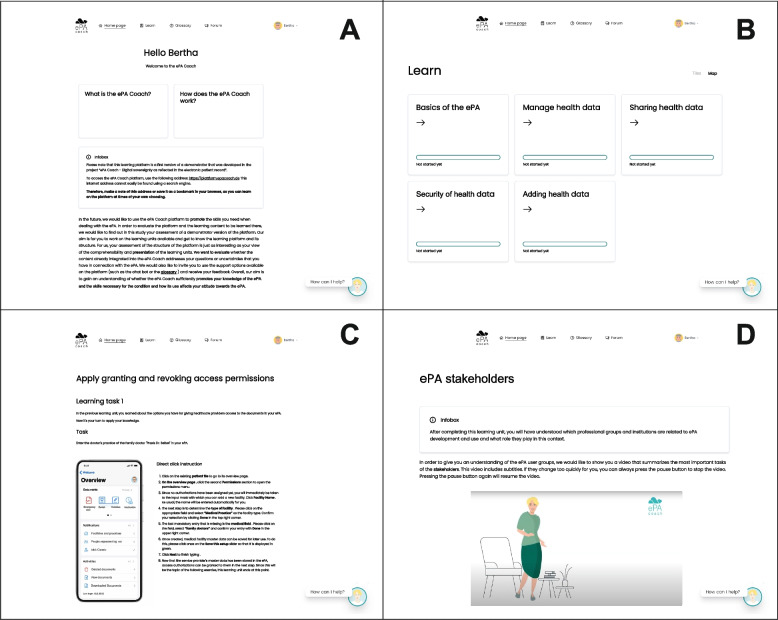
Translated screenshots of the ePA Coach learning platform: Landing page with introduction (**A**), Overview of the available learning topics (**B**), Exemplary learning unit with interactive ePHR mockup (**C**), Exemplary learning unit with video content (**D**)

Within the research project, “ePA Coach”, we developed a high-fidelity prototype of a learning platform as an empowerment tool for self-sufficient ePHR usage [39]. Since the platform was targeted toward OA, a participatory research approach was employed to investigate the general needs and preferences of OA regarding an empowerment tool through iterative surveys and tests [27, 40]. We used the results of our research to guide the development process and the design of the learning platform. The Octalysis framework served as the foundation for designing gamification elements to motivate users and was adapted based on insights from previous user studies [41, 42]. This included the identification of motivational core drives for promoting adherence and active learning. A micro-learning approach was adopted to ensure the effective acquisition of competencies required for confident ePHR use. The educational approach of the learning platform was based on the European Union’s DigComp framework [43]. This framework helped identify and incorporate the key competencies and learning requirements we needed to include in the learning platform. We used the framework, adapted it considering the preferences of OA, and used the resulting framework for the learning content creation process. The learning units were divided into three levels: “beginner”, “advanced” and “expert”. Each level addressed a different didactic concept [42]. The content classification was based on complexity, as well as on the cognitive dimensions to be addressed [39]. The learning units within the ePA Coach platform were presented in a multimedia format, offering both video and text content. In total, 32 learning units were available, covering five basic competencies. Each learning unit had an estimated completion time of approximately six to eight minutes. User progress was saved from one session to another. The learning units featured interactive elements that facilitated learning and encouraged users to practice what they had learned. Interactive mockups of an ePHR enabled users to practice navigation of the ePHR and perform specific tasks without using their actual health data. These mockups were included in various learning units. Additional interactive elements, such as exercises and tips, were incorporated to enhance learning motivation. Terms that may not have been familiar to the OA audience were explained in a glossary. Additionally, participants had the opportunity to ask other users questions about the ePHR or to answer questions posed by their peers through a forum on the learning platform, which was moderated by the study staff. Screenshots of the learning platform are shown in Fig. 2.

The available learning content covered the following overarching topics:

1. *Basics of the ePHR*: Basic information and access to the ePHR—Understanding the concepts of the ePHR (nine learning units).
2. *Managing health data*: Handling health-related information and data—Managing data, information and digital content stored in the ePHR (six learning units).
3. *Adding health data:* Adding health information to the ePHR—Digitizing documents, assigning metadata (six learning units).
4. *Sharing health data:* Sharing health-related data within the ePHR—Appointing deputies, managing access permissions (five learning units).
5. *Security of health data*: Information on the security of health data in the ePHR (six learning units).

### Chat-based learning assistant

**Fig. 3:**
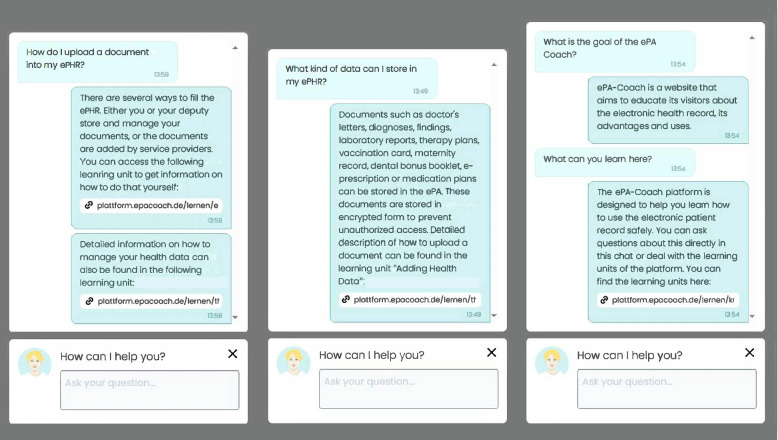
Exemplary interactions with the chat-based learning assistant

A learning companion in the form of an always-available chat-based assistant was integrated into our e-learning platform to help users with questions related to platform functionality or regarding the ePHR in general (e.g., “Where do I find information on data protection within the ePHR?” or “What is the ePHR?”, exemplary interactions are shown in Fig. 3). The assistant was available at the bottom right corner of each screen. To build the dialogue model of our learning assistant, we used the open-source version of the RASA Conversational AI software [44]. We provided ‘intents’ (possible user-intents during conversation) and several ‘responses’ that could be given in response to each ‘intent’. We also created so-called ‘stories’, which provide typical dialog flows as data for the dialog model for training purposes. This included questions on how to use the platform, the definition of various glossary items related to the ePHR, questions regarding the ePHR in general and reactions to chitchat questions. Additionally, we added questions and answers from gematik’s FAQ [45]. gematik is a German agency providing a regulatory framework for the ePHR and collects key questions regarding the ePHR and its use on the website. Overall, we integrated 107 intents, using 1439 example questions and 217 responses, into the dialogue model.

### Materials

#### Knowledge evaluation

In accordance with Baartman and de Bruijn [24], we aimed to evaluate the effects of the e-learning platform on the competence of OA when using the ePHR, by looking at the three factors, namely, skills, knowledge and attitude. To measure the change in the study participants’ knowledge of the ePHR and related information, a questionnaire was used during visit 1 and visit 2. The aim was to measure a change in topic-related knowledge. For this purpose, a questionnaire, consisting of 12 questions on general knowledge related to the ePHR, was created. This creation was based on a guide by Krebs [46] which focused on testing using this question format. The 12 questions used were designed as single-choice questions with four response options (see Supplementary Material 1). To ensure comparability, we used the same questions at baseline and follow-up. There was no feedback on the correctness of the responses given after visit 1 was completed. We changed the order of the questions for visit 2. Regarding the scoring, correct answers were given a point and incorrect answers were given 0 points. Thus, a score of 0 to 12 points was achievable in each questionnaire.

#### Skill evaluation

In order to determine not only the change in knowledge but also whether the use of the learning platform had an influence on practical skills regarding ePHR use, hands-on tasks were used in the form of mockups in both visits. Three tasks involving the operation of the ePHR were presented in the form of interactive ePHR mockups. The mockups thereby represented a version of a German ePHR. A password was hidden within the mockup provided for each task, which the study participants had to search for. The password was only displayed correctly if the task was completed successfully. As the main variable for skill evaluation, the time for completion of the three tasks was measured.

Additionally, subjects who were unable to complete the assignment at specific stages were also given support in the form of instructions. A strict protocol was developed for this purpose, in which it was precisely defined which hints could be given by the study personnel after a certain period of time. An additional scoring system was developed to evaluate the number of hints given for the skill evaluation (score for independent task performance). Each task within the ePHR mockup was divided into several steps. Two points were awarded if the respective step could be solved without hints. If a segment had to be supported with a hint, one point was awarded, and 0 points were given if a segment was not solved. As the tasks varied in complexity, six points were awarded for skills test one, 18 for skills test two and eight for skills test three. This resulted in a maximum score of 32 points.

**Fig. 4:**
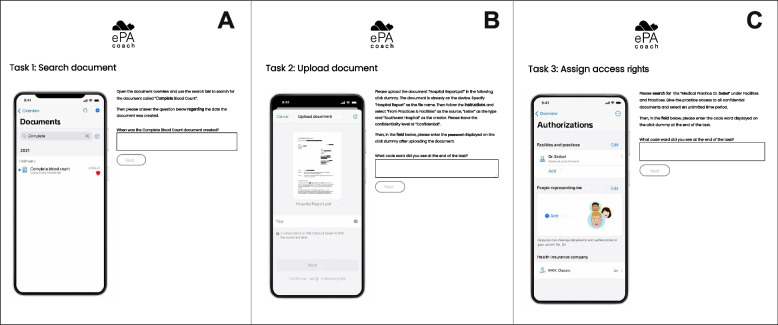
Screenshots of the tasks within the skills test: Finding a document within the ePHR (**A**), Uploading a document into the ePHR (**B**), Assigning access rights to a physician (**C**)

The tasks within the skill evaluation were:

- Searching for a document in the ePHR and identifying a relevant piece of information (Fig. 4, A)
- Adding a new document to the ePHR and providing metadata (Fig. 4, B)
- Assigning access rights to a specific service provider (Fig. 4, C)

#### TUI—post: ITU-subscale

In our study, we included the TUI developed by Kothgassner [36], specifically focusing on the ITU subscale in order to measure attitudes toward the ePHR. This subscale assessed participants’ intention and willingness to use a specific technology, providing valuable insights into their attitudes toward adopting new technology. Participants filled out the questionnaire by indicating their level of agreement with a series of statements. The responses are generally given by using a five-point Likert scale. The ITU subscale uses a visual analog scale with the maxima 0 (“I agree”) and 100 (“I do not agree”). The three items of this scale are used to calculate a score ranging from 0 to 300, with a higher number indicating a higher ITU. A principal component analysis with orthogonal rotation (Varimax with Kaiser normalization) was conducted by the authors of the assessment for the elderly subgroup of their study, revealing moderate sampling adequacy (KMO = 0.67) and significant Bartlett's test results (χ^2^ (276) = 865.95; *p* < 0.001), indicating sufficient inter-item correlations. Internal consistency (Cronbach's alpha) for the overall TUI questionnaire was assessed for both subgroups (younger vs. older participants). The internal consistency ranged from α = 0.66 to 0.89 across all samples, indicating good reliability for the overall construct.

#### SUS

We used the validated short questionnaire, SUS, to evaluate the usability of the ePA Coach learning platform [47, 48]. The SUS is an established questionnaire used in various usability studies and contains 10 items. Responses are given on a five-point Likert scale (1 = ‘strongly disagree’, 5 = ‘strongly agree’). The total score ranges from 0 to 100 (perfect usability). We included the validated German version in our study [49]. The authors report a consistent scale reliability and validity for the German-language assessment, with a Cronbach’s alpha greater than 0.80, and significant coefficient alpha values ranging from *r* = 0.54 to 0.74 for reliability.

#### CUQ

We used the CUQ to assess the usability of the chat-based learning assistant we integrated into the ePA Coach platform [38]. Answers were given (analog to the SUS) on a five-point Likert scale (1 = ‘strongly disagree’, 5 = ‘strongly agree’) and the total score was calculated out of 100. The CUQ was published in English. Two independent researchers, who were not part of the study personnel, translated the questionnaire into German. The study participants were able to specify whether they had used the chatbot on the learning platform. Only participants who had interacted with the chatbot filled out the questionnaire. The CUQ demonstrated good construct validity, as it successfully differentiated between chatbots with varying levels of usability (*p* < 0.05). Intra-rater reliability was supported by strong correlations (*r* > 0.7) between participants' assessments of the same chatbots two weeks apart [50].

#### Usage behavior of the learning platform

In order to analyze the usage behavior of the study participants during the intervention period, logging data were recorded and subsequently analyzed. These data included the number of logins within one week of use, the number of unique learning units completed and the total time of use. The time of use was measured as the time between the login and logout of each session (if no logout was performed, the last interaction with the e-learning platform for each day was used for the purposes of calculation).

### Data analysis

The quantitative data from the questionnaires were collected on paper and transferred to SPSS (IBM SPSS statistics version 27; IBM Corp., Armonk, NY, USA, 2020) for analysis. Only those participants who had completed at least three learning units within the ePA Coach were included in the analysis (all participants were thus included). For analysis of the usage behavior of the learning platform, the logging data of the participants were exported and integrated into the dataset.

Questionnaire data and logging data were analyzed descriptively and using inductive statistics. The Kolmogorov–Smirnov test and the Shapiro–Wilk test were performed to test for normal distribution. In the absence of normal distribution, the Wilcoxon test was used for within-group comparisons and the Mann–Whitney U test was used for between-group comparisons. We also used Fischer’s exact test for comparing study population characteristics. If normal distribution was present, a t-test was performed. Effect sizes were calculated using Pearson’s r. We reported median values (with interquartile range) and mean values (with standard deviation). To examine the correlations between variables, the Spearman correlation was calculated. We employed the standard alpha level of 0.05 for the interpretation of research findings and to counteract the problem of multiple testing, the Bonferroni correction for the alpha level was applied in the analysis of the two primary endpoints (comparison of the skill and knowledge test results in the intra-group comparison) [51]. This resulted in an alpha level of 0.025 for the interpretation of the primary endpoints.

## Results

### Study population description

**Fig. 5:**
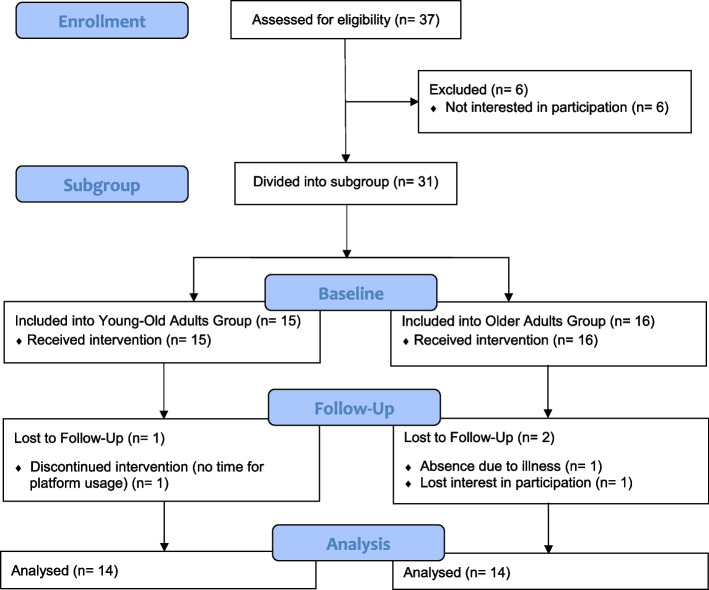
Flow Diagram according to Schulz et al. [52]

Table 1 shows the demographic characteristics of the individuals participating in both study groups. A total of 28 participants were included in our study and were assigned to one of the two subgroups based on age. Each arm included 14 participants (Flow Diagram in Fig. 5). The YOA had a mean age of 56.86 years while the OA had a mean age of 75.15 years. Both study groups included more women than men (YOA: 78.57%, OA: 57.14%) and all participants had a relatively high level of education. The YOA had more experience of using e-learning services and applications in the past. Of the YOA, 85.71% reported prior experience of e-learning, while only 50% of the OA had prior experience. Twelve of the 14 OA had prior knowledge of the ePHR, while only eight of the YOA knew about the ePHR prior to study participation. Only one of the YOA reported having practical experience of using the ePHR prior to the start of the study.

**Table 1:** Demographic characteristics of the study participants

	YOA	OA	*p*-value
Number of Participants [n]	14	14	
Sex (male/female) [n]	3/11	6/8	0.420^a^
Age (M (SD)) [years]	56.86 (4.24)	75.15 (5.43)	
Age Range (min. – max.) [years]	51—64	68—86	
TICS (M (SD))	37.50 (2.38)	35.71 (2.73)	0.076^b^
*Highest Education Level [n]*			.872^c^
Intermediate School	1	2	
Grammar School	4	2	
University of Applied Sciences	1	2	
University	8	7	
Not Specified	0	1	
*Marital Status [n]*			0.015^c^*
Single	1	0	
Married	10	5	
Divorced	3	4	
Widowed	0	4	
Not Specified	0	1	
Smartphone Usage (yes/no) [n]	14/0	12/2	0.481^a^
*Smartphone Usage [n]*			0.124^c^
I don’t have one	0	2	
I never use it	0	0	
I rarely use it	0	0	
I use it often	2	5	
I use it very frequently	12	7	
Prior Experience of E-Learning (yes/no) [n]	12/2	7/7	0.103^a^
Prior Knowledge of the ePHR (yes/no) [n]	8/6	12/2	0.209^a^
Prior Experience of Using the ePHR (yes/no) [n]	1/13	0/14	1.000^a^

*Abbreviations*: *YOA* Young-Old Adults, *OA* Older Adults, *M* Mean, *SD* Standard Deviation, *TICS* Telephone Interview for Cognitive Status, *ePHR* electronic Personal Health Record

^*^p ≤ .005

^a^Fischer’s exact test

^b^Unpaired t-test

^c^Mann-Whitney U test

### Usage behavior relating to the ePA Coach platform

The YOA used the e-learning platform, ePA Coach, for around six hours and 15 min on average, while the OA used the platform for a mean time of 14 h and 41 min (Table 2). With a mean of 5.71, the OA recorded significantly more logins during the one-week intervention period than the YOA, who had a mean number of logins of 3.86 (*U* = 50.00, Z = −2.227, *p* = 0.026). Meanwhile, both study groups completed almost the same average number of learning units on the learning platform during the period of use. With a mean completion of 24.93 (YOA) and 24.50 (OA) learning units, both groups completed almost 80% of the learning units provided on the platform.

**Table 2:** Usage behavior on the ePA Coach learning platform during the one-week intervention period

	YOA	OA	*p*-value
Total Usage Time (M (SD)) [minutes]	375.52 (253.99)	881.28 (843.57)	0.073
Number of Logins (M (SD))	3.86 (1.92)	5.71 (2.13)	0.026*
Number of Learning Units Completed (M (SD))	24.93 (12.79)	24.50 (12.02)	0.889

*Abbreviations*: *YOA* Young-Old Adults, *OA* Older Adults, *M* Mean, *SD* Standard Deviation

Mann–Whitney U test was performed

^*^p ≤ 0.05

During the intervention period, nine participants reported to have used the chatbot and filled out the CUQ (four YOA and five OA). A total number of 130 interactions with the chatbot were recorded. The mean CUQ score for these nine participants was 53.1 (*SD* = 17.68). The YOA had a mean CUQ score of 64.1 (*SD* = 10.90) and the OA of 44.4 (*SD* = 17.90). The scores for the CUQ can be interpreted analogue to SUS scores and indicate a “poor” or “not acceptable” usability for the OA and a “high marginal” or “OK” usability for the YOA [37].

### Results of the knowledge test

**Fig. 6:**
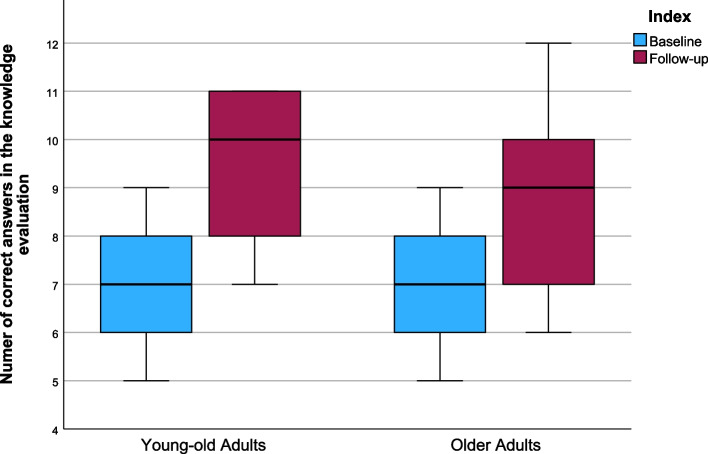
Boxplots of the number of correct answers in the knowledge evaluation with maximum and minimum values

When analyzing the results of the knowledge test (Table 3), it was evident that both the OA and the YOA had significantly more correct answers in the single choice test during the follow-up evaluation than during the baseline assessment (OA: *Z* = −2.339, *p* = 0.019, YOA: *Z* = −3.219. *p* = 0.001). Out of the 12 single-choice questions in the baseline assessment, the OA reached a median value of 7.00 (*IQR* = 2.00, *M* = 7.14, *SD* = 1.35) correct answers, while the YOA also had 7.00 (*IQR* = 2.00, *M* = 6.86, *SD* = 1.41) correct answers. In the follow-up evaluation, the median value of correct answers was 9.00 (*IQR* = 3.00, *M* = 8.57, *SD* = 1.65) for the OA and 10.00 (*IQR* = 3, *M* = 9.36, *SD* = 1.49) for the YOA. The effect was large in both groups (*YOA*: *r* = 0.86, *OA*: *r* = 0.63). The median values for both groups as a boxplot are shown in Fig. 6. When comparing the values for the mean differences of correct answers between the two groups, no significant difference was present in the knowledge test between the baseline and follow-up assessments (*U* = 64.50, *Z* = −1.586, *p* = 0.125) (Table 4).

**Table 3:** Comparison of pre- and post-results of outcome variables

	Group	Baseline: *Mdn (IQR)*	Follow-up: *Mdn (IQR)*	*p*-value	*r*
*Knowledge test [number of correct answers]*	All study participants	7.00 (2.00)	9.00 (3.00)	< 0.001**	0.75
	YOA	7.00 (2.00)	10.00 (3.00)	0.001**	0.86
	OA	7.00 (2.00)	9.00 (3.00)	0.019**	0.63
*Skill test [in seconds]*	All study participants	588.00 (332.00)	438.50 (203.00)	< 0.001**	0.76
	YOA	487.00 (110.00)	351.00 (175.00)	0.012**	0.67
	OA	746.50 (256.00)	539.00 (186.00)	0.002**	0.82
ITU score	All study participants	282.00 (46.00)	262.00 (122.00)	0.038*	0.39
	YOA	279.00 (33.00)	255.00 (193.00)	0.132	0.40
	OA	284.00 (53.00)	262.00 (109.00)	0.126	0.41

*Abbreviations*: *YOA* Young-Old Adults, *OA* Older Adults, *Mdn* Median, *IQR* Interquartile Range, *ITU* Intention To Use

Wilcoxon test was performed

*p* ≤ 0.05

^**^p ≤ 0.025

**Table 4:** Comparison of mean differences of outcome results between the YOA and OA group

	Group	*Mdn (IQR)*	*p*-value	*r*
*Knowledge test [number of correct answers]*	YOA	2.50 (1.00)	0.113	0.42
	OA	1.50 (3.00)		
*Skill test [in seconds]*	YOA	−152.50 (250.00)	0.168	0.37
	OA	−226.00 (299.00)		
ITU score	YOA	−4.00 (187.00)	0.535	0.17
	OA	−1.50 (34.25)		

*Abbreviations*: *YOA* Young-Old Adults, *OA* Older Adults, *Mdn* Median, *IQR* Interquartile Range, *ITU* Intention To Use

Mann–Whitney U test was performed

^*^p ≤ .05

### Results of the skills test

**Fig. 7:**
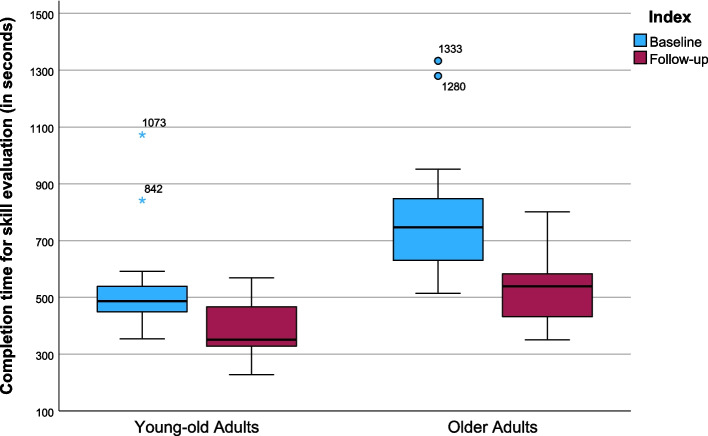
Boxplots of the total completion time for the skill evaluation with maximum, minimum and outliers

Similar results were observed regarding the time taken by the participants to complete the three interactive ePHR mockups as part of the skills test (Table 3). Here too, a significant difference was found in the completion time before and after the intervention in both groups, with both groups requiring less time to complete the tasks in the follow-up survey (OA: *Z* = −3.045, *p* = 0.002; YOA: *Z* = −2.512, *p* = 0.012). The median processing time for solving the three practical tasks was reduced from 746.50 s (*IQR* = 256.00, *M* = 797.00, *SD* = 248.70) to 539.00 s (*IQR* = 186.00, *M* = 538.36, *SD* = 139.44) for the OA and from 487.00 s (*IQR* = 110.00, *M* = 544.79, *SD* = 189.66) to 351.00 s (*IQR* = 175.00, *M* = 368.29, *SD* = 115.72) for the YOA (Table 4). A large effect size was also present here (OA: *r* = 0.82, YOA: *r* = 0.67). Figure 7 shows the median values for both groups in a boxplot. Similar to the results of the knowledge test, the differences in processing time in the pre-post comparison between the two groups were not significant (*U* = 173.00, *Z* = −1.378, *p* = 0.178).

In addition to the time required by both groups to solve the skills test, we also analyzed the scoring for these tasks. The independent task performance score was assigned according to the number of hints needed for solving the tasks. In the YOA group, a median of 32.00 (*IQR* = 2.00, *M* = 30.88, *SD* = 1.29) out of 32 points was achieved in the baseline evaluation. The YOA achieved a median score of 32.00 (*IQR* = 2.00, *M* = 30.93, *SD* = 1.73) in the follow-up survey. There was no significant difference between the baseline and follow-up scores achieved (*Z* = −0.574, *p* = 0.566). The OA achieved a median score of 29.00 (*IQR* = 3.00, *M* = 29.29, *SD* = 1.77) in the baseline test and 28.50 (*IQR* = 3.50, *M* = 28.00, *SD* = 2.75) in the follow-up survey. Here, too, there was no significant difference between the two measurement points (*Z* = −1.485, *p* = 0.138). Furthermore, when comparing the mean difference between the groups, no significant difference was found (*U* = 67.00, *Z* = −1.463, *p* = 0.144).

### ITU

When looking at the results from the ITU subscale of the TUI, no significant difference in the ITU the ePHR was found between the baseline and follow-up assessments either within the groups or between the groups (Tables 3 and 4). The only significant difference was found when comparing the baseline and follow-up scores of all study participants (*Z* = −2.074, *p* = 0.038). The participants of both groups reported a median ITU score of 282.00 (*IQR* = 46.00, *M* = 259.29, *SD* = 63.06) during the baseline evaluation and a score of 262.00 (*IQR* = 122.00, *M* = 223.11, *SD* = 82.31) in the follow-up evaluation.

### SUS

The mean SUS score for the usability evaluation of the ePA Coach learning platform was 64.04 (*SD* = 19.57, *Mdn* = 70.00, *IQR* = 24.40) for all study participants. The YOA reported a mean usability rating of 68.21 (*SD* = 16.80, *Mdn* = 72.50, *IQR* = 10.60) and the OA, 59.86 (*SD* = 21.81, *Mdn* = 56.25, *IQR* = 34.90). No significant difference was present when comparing the scores between the two groups (*U* = 70.500, *Z* = −1.267, *p* = 0.205). The reported scores for both groups indicate a “OK” or “high marginal” perceived usability of the learning platform according to Bangor [37].

### Correlation of outcome variables with usage time and number of learning units completed

The correlation between the results of the knowledge test, the skills test and the measured ITU the ePHR (mean differences between baseline and follow-up evaluations) was analyzed in relation to the usage behavior on the learning platform (Table 5). On the one hand, the correlation of the outcomes with the total usage time of the ePA Coach learning platform by the study participants was examined. On the other hand, the correlation of the assessments with the number of completed learning units was calculated. None of the calculated correlations showed significant results.

**Table 5:** Correlation of outcome variable mean values with total usage time and number of learning units completed

	Group	*df*	*p*-value	*r*_*s*_
*Correlation of outcome variables with total usage time*				
* Knowledge test [number of correct answers] – total usage time [in seconds]*	All study participants	26	0.239	0.23
	YOA	12	0.556	0.17
	OA	12	0.850	0.48
* Skill test [in seconds] – total usage time [in seconds]*	All study participants	26	0.996	-0.00
	YOA	12	0.288	0.31
	OA	12	0.681	-0.12
ITU score – total usage time *[in seconds]*	All study participants	26	0.281	0.21
	YOA	12	0.182	0.38
	OA	12	0.998	-0.00
*Correlation of outcome variables with the number of learning units completed*				
* Knowledge test [number of correct answers] – completed learning units*	All study participants	26	0.128	0.30
	YOA	12	0.239	0.34
	OA	12	0.426	0.23
* Skill test [in seconds] – completed learning units*	All study participants	26	0.849	-0.04
	YOA	12	0.559	0.17
	OA	12	0.420	-0.24
* ITU score – completed learning units*	All study participants	26	0.941	0.02
	YOA	12	0.439	0.23
	OA	12	0.238	-0.34

*Abbreviations*: *YOA* Young-Old Adults, *OA* Older Adults, *df* degrees of freedom, *ITU* Intention To Use

Spearman correlation was calculated

^*^p ≤ 0.05

### Correlation of outcome variables with SUS

We calculated the correlation between the SUS score and the mean differences (baseline and follow-up) of the outcome variables (Table 6). No significant correlation was present between the SUS and the results of the knowledge test and the ITU score, either for all study participants or for one of the two study groups. A significant correlation, with a strong effect size, was present between the SUS scoring and the mean difference in the completion time of the skill evaluation among the group of OA (r_s_(12) = −0.60, *p* = 0.025). Furthermore, a significant correlation with a moderate effect size can be observed between the SUS score and the mean difference in the number of hints given during the skill evaluation, when analyzing the results of all study participants (r_s_(26) = 0.40, *p* = 0.036).

**Table 6:** Correlation of outcome variable mean values with SUS

	Group	*df*	*p*-value	*r*_*s*_
*Knowledge test [number of correct answers]—SUS*	All study participants	26	0.284	0.21
	YOA	12	0.420	0.24
	OA	12	0.908	0.04
*Skill test [in seconds]—SUS*	All study participants	26	0.452	-0.15
	YOA	12	0.916	0.03
	OA	12	0.025*	-0.60
*Independent task performance—SUS*	All study participants	26	0.036*	0.40
	YOA	12	0.487	-0.20
	OA	12	0.096	0.46
ITU score—*SUS*	All study participants	26	0.753	0.06
	YOA	12	0.631	0.14
	OA	12	0.747	0.10

*Abbreviations*: *YOA* Young-Old Adults, *OA* Older Adults, *df* degrees of freedom, *SUS* System Usability Scale, *ITU* Intention To Use

Spearman correlation was calculated

^*^p ≤ 0.05

## Discussion

### Primary outcomes

The results of the pilot trial highlighted a significant increase in knowledge and a decrease in time in accomplishing the skill tasks using the ePHR mockups within both groups. The faster completion of the tasks can be considered as an improvement in efficiency and, therefore, skill across both groups. No significant differences in both knowledge and skill evaluation were found between the groups. This suggests that both groups benefited equally from the training provided by the e-learning platform, and there were no age-related disparities in outcomes. Since there was no confirmation of the correctness of the answers given in the knowledge test during the baseline visit and the order of the questions was changed for the follow-up visit, it can be assumed that the retest effects had been minimized for the knowledge test. As the skill tasks were tasks consisting of multiple steps, a reduction of the retest effect can also be assumed here.

The dearth of existing publications regarding tools to improve digital literacy in the use of ePHRs, particularly among the older age group, highlights the need for further research and emphasizes the novelty of our findings. One notable exception is a study by Nahm et al. [23], who used a theory-based patient-portal e-learning program among OA with chronic illnesses. A subtopic of module 1 in their program included learning content relating to the “electronic health record and personal health record”. In their randomized, controlled study, the intervention group significantly improved their patient-portal knowledge after three weeks. The effect size of our pilot trial for knowledge acquisition was 0.63 within the older age group, which was almost identical to the effect size of 0.62 reported in the study carried out by Nahm et al. However, their data were collected exclusively on the basis of self-reports in online surveys; a skills test was not part of their evaluation. A study by Taha et al. [53] assessed the ability of middle-aged adults (40–59 years) and OA (60–85 years) to apply routine health management tasks to a simulated ePHR. Both age groups experienced significant difficulties in using the PHR to complete the tasks.

Previous research has demonstrated the potential for computer-based interventions to enhance eHealth literacy among OA. Xie [54] showed in her study that computer training, using the online resources of two National Institutes of Health, significantly improved OA’ computer and web knowledge, which is crucial for eHealth literacy. Manafò et al. [55] demonstrated that OA improved their perceived eHealth literacy skills after using the eHealth literacy tool, eSEARCH. A meta-analysis by Dong et al. [25] on the effectiveness of digital health literacy interventions in OA revealed that such interventions can have a significant effect on OA’ knowledge of computers, the Internet and patient portals. Among the studies included in the meta-analysis, only two studies assessed skills for computer and internet use based on procedural tests. However, the pooled effect of skills was not statistically significant, in contrast to our results of the pre-post comparison of the ePHR skills test completion time in both groups. In the interventions analyzed in the meta-analysis, the effect on computer and Internet skills was tested. However, our study relating to skill development focused more on a specific use case, which was the application of the ePHR. In contrast to other interventions, we were also able to offer interactive ePHR mockups within the platform, which could explain the improvement in skill. At the beginning of the project, a requirements analysis revealed that the participating OA had limited knowledge about the German version of the ePHR and faced challenges in obtaining sufficient information. The analysis showed a high demand for learning content on essential information, as well as for competencies related to the ePHR [27]. The results of this study are promising and show an initial efficacy in knowledge gain and skill acquisition.

### Secondary outcomes

In our present study, the results indicated no significant difference in the ITU the ePHR in both groups. However, a significant decrease in the TUI within the domain of ITU was observed in the overall sample. It should be noted that the ITU score remained in the upper range of the scale. Since almost no practical experience in using the ePHR was present within our study sample prior to participation, we assume that the use of our e-learning platform led to an awareness among participants regarding the processes and implications associated with the German ePHR and caused a certain degree of restraint. We, therefore, believe that the post-interventional results are associated with a more informed decision about the ITU compared to the baseline assessment.

Lober et al. [56] elucidated that the OA’ adoption of ePHRs is primarily hindered by their limited computer and health literacy. The relevance of eHealth literacy was also demonstrated by the cross-sectional survey of OA with hypertension or diabetes, which examined relationships between patient-portal usage and eHealth literacy. Among the OA the eHealth literacy was positively associated with portal usage and interest in health-tracking tools [57]. However, our results show that a significant improvement in knowledge and skills does not necessarily lead to a higher intention of future use of an ePHR, although this may be a prerequisite for successful usage. Logue and Effken [58] found that other factors, such as personal attributes, environmental conditions, technological aspects, chronic illnesses and behavioral elements simultaneously act as obstacles and/or enablers for OA with chronic illnesses in adopting PHRs.

Furthermore, no significant differences were observed in the number of completed learning units, indicating comparable usage behavior between the two groups in our study. The only notable observation was the significant difference in the number of logins within a training week, with seniors logging in significantly more often (5.71 logins) than the younger age group. This indicates that OA distributed their usage over multiple sessions. Although no significant difference was present, it should be mentioned that the OA spent more than twice as much time on the learning platform. With an identical number of units completed, it can be assumed that the OA were slower in completing the learning units. The difference in results between the knowledge and skills test did not significantly correlate with the usage behavior on the learning platform. One possible explanation is that participants extensively engaged with the learning platform over the course of one week, resulting in minimal differences between participants.

The results of the usability analysis using the SUS showed a mean score of 64.04 across the overall sample. There were no significant differences between the two age groups when analyzing the findings. The total score of the SUS can be interpreted as “OK” or “high marginal” according to Bangor [37]. Contrary to our expectations and despite the desired improvements, the usability was rated slightly lower than in the previous study involving the first prototype of the ePA Coach learning platform [28]. There are several reasons why usability could be rated at this level. In the previous online intervention study, the first prototype was criticized for its complexity. With the addition of learning units, the platform has now become even more complex, which could explain the lower usability rating in the current study. In a future study, the intervention period could be expanded, although issues of adherence should be addressed accordingly. The results of the CUQ and the few interactions with the chatbot indicate low chatbot usability, which could also have lowered the overall usability score of the SUS. Although usability was high marginal, there was no restriction on knowledge gain and skill acquisition, as the results of these outcomes were still significant. An improved usability of the platform may improve the skills acquisition of OA, in particular, and the results of the correlation between the mean difference of the completion time in the skills test and the SUS score show a strong negative correlation in OA.

Starting in 2025, the number of registered ePHRs will increase significantly in Germany, due to the introduction of an opt-out approach [59]. From this time onwards, all those with statutory health insurance in Germany will be faced with the decision of whether to reject or accept an ePHR and, if they accept, they may encounter possible comprehension and operating problems. Our findings show that an increase in the competence of YOA and OA when using an e-learning platform is possible if competence is defined by skills, knowledge and attitude. Participants in both groups benefited from the use of the learning platform in terms of their knowledge of the ePHR and their skills in relation to its operation. Our outcomes suggest that the attitude, which we referred to as the ITU the ePHR, did not differ significantly in both groups. However, further research is needed in the near future to validate the findings of our pilot trial and to investigate the further potential benefits and the effectiveness of such interventions. Therefore, future studies should investigate the application over a longer period of time.

### Limitations

In our study, we investigated the effects of an innovative e-learning platform with interactive elements for empowering the use of the ePHR. The impact on OA’ knowledge and skills were measured for the first time regarding the German ePHR. Positive effects were demonstrated in both age groups.

The pilot trial presented has some limitations that should be considered when interpreting the results. Due to the exploratory pilot character, there was no a priori sample size calculation. The statistical significance of the analysis is limited with such a small sample size, which can increase the risk of a type II error. A future study for evaluating the effectiveness of ePA Coach should be carried out with a larger sample size, and designed as a randomized, controlled trial to achieve even more robust and generalizable results. The lack of blinding may have introduced expectation bias, observer bias, and/or detection bias. Our study did not have a balanced gender distribution and included predominantly highly educated adults. A larger study with suitable stratification techniques could counteract this potential bias and could also help identify other subgroups that could benefit from the use of the learning platform (such as other age groups or stratification by digital competencies). Regarding the primary outcomes (knowledge and skill), no validated assessments were used. To our knowledge, there are currently no validated assessments for testing competence in the use of ePHRs. In addition, the knowledge and skills tests were identical during both visits (baseline and follow-up evaluations). Even if some measures were taken to reduce this (no clarification of the knowledge question results and changing the order of the questions, development of multi-step solution paths for the competence tests), retest effects could still have been introduced if the participants remembered the solution to the tasks. Furthermore, the transferability of the participants’ competence gain to the actual use of the ePHR could not be investigated, as the implementation of the ePHR in Germany is still at an early stage. Due to the design of the ePA Coach as a website, only those with access to the internet and basic digital skills could be included in our study.

## Conclusion

After one week of using the ePA Coach e-learning platform young-old and older adults showed a significant increase in both ePHR knowledge and operation skills. Although the usability was perceived as high marginal, the platform was used extensively. Its effects on the study outcomes indicate that it has the potential to promote ePHR competencies. To accelerate the implementation of the ePHR into the German healthcare system, it may prove helpful to give older adults the opportunity to acquire competencies in specifically designed environments. In the future, this could be helpful in promoting the uptake of ePHR by the target group.

## Supplementary Information

Supplementary Material 1.

## Data Availability

The datasets used for this study are available from the corresponding author upon reasonable request.

## Abbreviations

ePA: Elektronische Patientenakte
ePHR: Electronic personal health record
ITU: Intention to use
IQR: Interquartile range
Mdn: Median
SD: Standard deviation
SUS: System Usability Scale
TICS: Telephone Interview for Cognitive Status
TUI: Technology Usage Inventory
OA: Older adults
YOA: Young-old adults
