# Supplementary material for: Evaluation of an e-learning platform promoting electronic personal health record competence: a pilot trial in older adults
Source: BMC Public Health. 2025 Mar 15;25:1016. doi: 10.1186/s12889-025-22242-0 (PMC11910860; doi:10.1186/s12889-025-22242-0)
Supplement: Supplementary file 1 — Supplementary Material 1. [file 12889_2025_22242_MOESM1_ESM.docx]

| **Knowledge Evaluation** | |
| --- | --- |
| **1.** | What kind of data can be managed within the electronic Personal Health Record? |
|  | Health related data and documents  Basic data of the insured person  Bank data  Accounting data  **Please select only ONE correct answer from the options.* |
| **2.** | Who is (among others) referred to as a “service provider” in the context of the statutory health insurance? |
|  | The gematik institution  Federal Ministry of Health  Hospitals  Research facilities  **Please select only ONE correct answer from the options.* |
| **3.** | The following information **cannot** yet be saved within your ePHR: |
|  | Treatment reports/doctor's letters  Emergency data  Electronic medication plan  Data from health applications  **Please select only ONE correct answer from the options.* |
| **4.** | The following login option is **not** common when accessing into an ePA: |
|  | Facial recognition (for example FaceID)  Insurance number  User name and password  Health card via an NFC-enabled smartphone and PIN  **Please select only ONE correct answer from the options.* |
| **5.** | Mr. Walther would like to find a doctor's letter in his ePHR which was uploaded by his cardiologist last spring. With which search term will he have **no** success in the search bar? |
|  | Name of a medication that is included in the doctor's letter  Author of the document  Date of upload  Medical discipline of the physician  **Please select only ONE correct answer from the options.* |
| **6.** | Ms. Müller would like to delete the data and documents of her stay in hospital from her ePHR. She notices that she seems to have agreed to numerous irrelevant documents being placed in her ePHR by the hospital. She deletes the documents from the ePHR, but she is afraid that these documents have now been deleted forever. What Ms. Müller does not know is that all documents that were created by service providers and added to her ePHR by the hospital are still available in... |
|  | … the hospital's database.  … the health insurance company's database.  … the database of her primary care physician.  … in the storage of her smartphone.  **Please select only ONE correct answer from the options.* |
| **7.** | Which of the following statements about metadata of documents (additional digital information about stored documents) in the ePA is **incorrect**? |
|  | Metadata are required to make documents in the ePHR easier to find.  Only your treating physician can specify metadata for your documents.  Some metadata need to be manually entered when uploading  documents to your ePHR.  Metadata can include information such as the medical context of the  creation, the type of document or the medical discipline of the creator.  **Please select only ONE correct answer from the options.* |
| **8.** | Mr. Müller has an old X-ray image at home that he would like to digitize and upload to his ePHR for future treatments. To do this, he takes a picture of the document with his smartphone. To ensure that the image is suitable for use in your ePHR, he makes sure that it meets certain criteria. Which of the following criteria does **not** play a role? |
|  | If possible, only the document and as little other background as  possible should be visible on the image.  The image quality should be sufficient to be able to easily see the  contents of the document.  The X-ray image should be shown as completely as possible.  The brightness of the image during capture is not important.  **Please select only ONE correct answer from the options.* |
| **9.** | Health-related information is considered to be personal data and is subject to special protection. When using the ePHR, all important information on the protection of personal and health-related data used in the context of the ePHR is specified in the data protection provisions for the ePHR. Which of the following statements about the privacy policy is true? |
|  | The data protection officer is named in the data protection policy.  Consent to the data protection policy is not required for the use of the  ePHR.  Once I have given my consent to the data protection policy, I cannot  withdraw it.  The health insurance company is not legally obliged to ensure the  protection of personal data.  **Please select only ONE correct answer from the options.* |
| **10.** | If you decide not to use the ePHR, the following applies: |
|  | Use of the ePHR is voluntary; you have the right not to use it.  Service providers may exclude you from care if you do not wish to use  the ePHR.  Service providers can create an ePHR for you without your consent.  Legally, the non-use of your ePHR may have a negative effect on your  healthcare provision.  **Please select only ONE correct answer from the options.* |
| **11.** | Mr. Walther would like to appoint a deputy to manage his ePHR on his behalf. Which of the following requirements must the deputy fulfill? |
|  | The deputy must have his or her own registered ePHR.  The deputy must be insured by the same health insurance company as  Mr. Walther.  The deputy must be related to Mr. Walther.  The deputy must be the legal guardian of Mr. Walther.  **Please select only ONE correct answer from the options.* |
| **12.** | You can assign so-called "confidentiality levels" to the documents within the ePHR, which can be used to determine who has access to the respective document. Who can assign these confidentiality levels without special authorization? |
|  | Your treating physician.  You yourself.  Your partner.  Your pharmacy.  **Please select only ONE correct answer from the options.* |
